# Successful Removal of a Biliary Stent in the Jejunum Using Double-Balloon Enteroscopy

**DOI:** 10.31661/gmj.v9i0.1809

**Published:** 2020-12-18

**Authors:** Arash Dooghaie Moghadam, Niloofar Razavi-Khorasani, Pegah Eslami, Sandra Saeedi, Ermia Farokhi, Bobak Moazzami, Azim Mehrvar, Shahrokh Iravani, Mahmood Reza Hashemi, Masoud Dooghaei Moghadam

**Affiliations:** ^1^Liver Transplantation Research Center, Tehran University of Medical Sciences, Tehran, Iran; ^2^Gastroenterology and Hepatobiliary Research Center, AJA University of Medical Sciences, Tehran, Iran; ^3^Research Center for Cancer Screening and Epidemiology, AJA University of Medical Sciences, Tehran, Iran; ^4^Digestive Diseases Research Institute, Tehran University of Medical Sciences, Tehran, Iran

**Keywords:** Double-Balloon Enteroscopy, Device Removal, Bile Duct, Stents, Cholangiopancreatography, Endoscopic Retrograde

## Abstract

**Background:**

Plastic biliary stent placement has been widely used as a safe approach for the management of hilar neoplasms or the dilation of benign biliary obstruction. Despite the complexity of this procedure, this approach is followed by a few complications. The incidence rate of stent migration is about 10%. In a majority of cases, the migrated stents are retained within the gastrointestinal tract and pass through the intestine with no complication or need for medical intervention.

**Case Report:**

In this paper, we described the case of the migrated biliary stent with prolonged abdominal pain, which was removed successfully by using double-balloon.

**Conclusion:**

In the case of patient with prolonged abdominal pain and previous history of biliary stent placement, migration of stent should be considered as differential diagnosis and Double-Balloon Enteroscopy can be a safe approach in those cases.

## Introduction

Since the introduction of biliary stent placement for the management of bile duct pathologies, there have been remarkable achievements in the field of therapeutic pancreaticobiliary endoscopy [1,2]. Biliary stenting has been routinely used to resolve obstructive jaundice using a non-surgical approach [3-5]. Although the use of these procedures has been accompanied with few complications, several adverse events (namely pancreatitis, cholecystitis, hemorrhage, cholangitis, and migration of biliary stent) following the stent placement are found in approximately 5–10% of cases [6,7]. The majority of these complications occur during or shortly after the procedure. Long-term complications such as stent migration are rare and less predictable, making the diagnosis more complicated. The incidence rate of stent migration is about 10% [8-10]. In a majority of cases, migrated stents are retained within the gastrointestinal tract and pass through the intestine with no complication or need for medical intervention. However, in rare cases, the migrated stent may get stuck in the duodenum and make endoscopic retrieval unfeasible. Double-balloon enteroscopy (DBE) is considered as an emerging endoscopic modality that enables direct visualization of the small bowel and successful removal of foreign bodies. These goals could not be achieved by using standard upper gastrointestinal endoscopy [11]. Herein, we present a successful case of the DBE procedure for the removal of a migrated biliary stent with prolonged abdominal pain.

## Case Presentation

**Figure 1:**
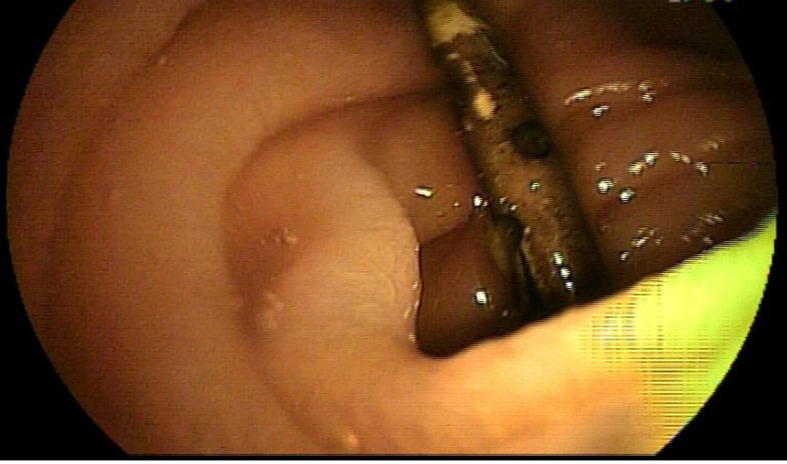
Double-balloon enteroscopy revealed a plastic biliary stent in the proximal jejunum with no endoscopic evidence of perforation

A 65-year-old man who suffered from chronic abdominal pain was admitted to our hospital for further evaluation. The patient described his symptoms as colicky pain without radiation. A significant weight loss was also observed in this patient. In another hospital, he was diagnosed with colon cancer three years ago and underwent colectomy. The patient had undergone chemotherapy for six months. At the same time, the patient was suffering from right upper quadrant abdominal pain and was diagnosed with multiple gallstones. He was a candidate for endoscopic retrograde cholangiopancreatography procedure; hence, a plastic stent was placed inside the common bile duct to provide biliary drainage. After six months, he referred once more with recurrent colicky abdominal pain not resolved by analgesics. On examination, his body temperature was 37.1°C, his heart rate was 86 beats per minute, his respiratory rate was 12 breaths per minute, and his blood pressure was 110/65 mm Hg. An abdominal examination revealed a soft and non-distended abdomen with no sign of tenderness. Moreover, no mass was palpable, and he had no rebound tenderness, guarding, or organomegaly. The rest of the physical examination revealed no remarkable finding. Laboratory results were also unremarkable. The evaluation performed at the outside hospital included upper gastrointestinal (GI) series, colonoscopy, abdominal radiograph, and ultrasonography. No abnormal finding was detected in all these tests. A computed tomography (CT) scan was performed after two months due to the prolonged abdominal pain. Radiological findings depicted the biliary stent dislocation and migration to the jejunum. The patient was then referred to a gastroenterologist to have DBE. He underwent DBE via the antegrade approach. In order to have sedation, Pethidine hydrochloride (25 mg), Midazolam (2 mg), and Propofol were used. Furthermore, we used Fujinon (Fujinon EN− 450T5, Fujinon, Tokyo, Japan) as an endoscope. As the endoscope was inserted, a “U-shape” plastic stent was found at the end of the jejunum (Figure 1). The snare wire was used for checking the possible adhesion and penetration of the stent into the intestinal lumen. When an endoscopist ensured that the stent was not attached to the lumen, the stent was gently retracted from jejunum with no complication. Moreover, severe adherent loops were observed at the distal jejunum site and caused partial obstruction symptoms in this patient. The patient had a successful recovery and was discharged the same day. All the symptoms were recovered, and he did not experience similar abdominal pain one year after the procedure.

## Discussion

 Plastic biliary stent placement has been widely used as a safe approach for the management of hilar neoplasms or the dilation of benign biliary obstruction. Despite the complexity of this procedure, this approach is followed by a few complications. Nevertheless, long-term complications, such as stent migration, can be problematic in the diagnosis procedure. Both proximal and distal migration is rare and less commonly reported by other investigators. There is also limited data regarding the endoscopic retrieval methods for stents migrated proximally or distally. DBE has recently gained considerable attention and is proved to be effective in the management of small-bowel disorders such as the retrieval of a foreign object in the small intestine. In this case report, we encountered a rare condition in which a biliary stent migrated into the jejunum. We successfully extracted a plastic biliary stent, which was kept within jejunal loops and caused prolonged abdominal pain and partial obstruction in the patient. In the literature, several retrieval methods, including polypectomy snare, Dormia basket, endoscopic suture-cutting device, and ‘hot’ biopsy forceps, are introduced to extract individual wire filaments. In this case, we used snare wire for stent removal. No complication was observed during the stent removal procedure. In summary, the distal migration of stent causing a partial obstruction should be concerned in order to make clinical decisions for patients with prolonged abdominal pain and a history of previous biliary stent placement. Moreover, DBE offers a safe therapeutic endoscopic approach and can be used as a first-line treatment in cases with stent migration.

## Conclusion

 In summary, the distal migration of stent causing a partial obstruction should be concerned in order to make clinical decisions for patients with prolonged abdominal pain and a history of previous biliary stent placement. Moreover, DBE offers a safe therapeutic endoscopic approach and can be used as a first-line treatment in cases with stent migration.

## Conflict of Interest

 There are no conflicts of interest to declare.
